# Synchronous Hepatic Neuroendocrine Carcinoma and Rectal Adenocarcinoma With Pontine Metastasis: A Diagnostic Dilemma

**DOI:** 10.7759/cureus.86100

**Published:** 2025-06-15

**Authors:** Ebram Said, Yasmin Gerais, Mohamed Boshnaf, Anas Mahmoud, Hossam Elbenawi, Ahmed Salem, Michelle OLeary, Sammy Hamad, Bachar Hamad

**Affiliations:** 1 Internal Medicine, Ascension St. Joseph Hospital, Chicago, USA; 2 Gastroenterology, Saint Joseph Medical Center, Joliet, USA; 3 Internal Medicine, University of Benghazi, Faculty of Medicine, Benghazi, USA; 4 Internal Medicine, St. Joseph’s University Medical Center, Paterson, USA; 5 Internal Medicine, Mansoura University School of Medicine, Mansoura, EGY; 6 Internal Medicine, Maimonides Medical Center, New York, USA; 7 Pathology, Saint Joseph Medical Center, Joliet, USA

**Keywords:** brain metastasis pontine, gastrointestinal neuroendocrine tumor, mixed adeno-neuroendocrine carcinoma, multiple primary malignancies, rectal malignancy, s: synchronous primary tumors

## Abstract

We present a complex case of a 53-year-old male with stage IV large-cell neuroendocrine carcinoma (NEC) of unconfirmed primary origin, manifesting with a pontine brain mass, multiple hepatic lesions, lymphadenopathy, and a synchronous rectal adenocarcinoma. Imaging and pathology revealed distinct morphologic and immunohistochemical profiles: hepatic biopsy confirmed large-cell NEC positive for synaptophysin and CD56, whereas rectal biopsy showed adenocarcinoma with intestinal markers and no neuroendocrine differentiation. Differential diagnoses included two independent primary malignancies, mixed adenoneuroendocrine carcinoma (MANEC), or colorectal adenocarcinoma with neuroendocrine differentiation in the liver. Although molecular profiling or clonal comparison was considered to clarify the primary origin, it was not performed due to clinical constraints. This report underscores the diagnostic challenges in distinguishing synchronous multiple primaries from metastatic or mixed tumors, with significant implications for staging and treatment.

## Introduction

Neuroendocrine carcinomas (NECs) are rare and aggressive neoplasms often presenting at an advanced stage with widespread metastases [1]. Colorectal adenocarcinoma is one of the most common gastrointestinal malignancies, but it rarely coexists with NEC in a synchronous pattern [2]. The presence of pontine brain metastasis further complicates the diagnostic picture. Determining whether such cases represent dual primary malignancies or a high-grade mixed histological tumor is vital, as it guides therapeutic decision-making. This distinction is crucial as it determines specific staging protocols, influences the selection of chemotherapy regimens, and affects the overall prognosis.

## Case presentation

**Figure 1:**
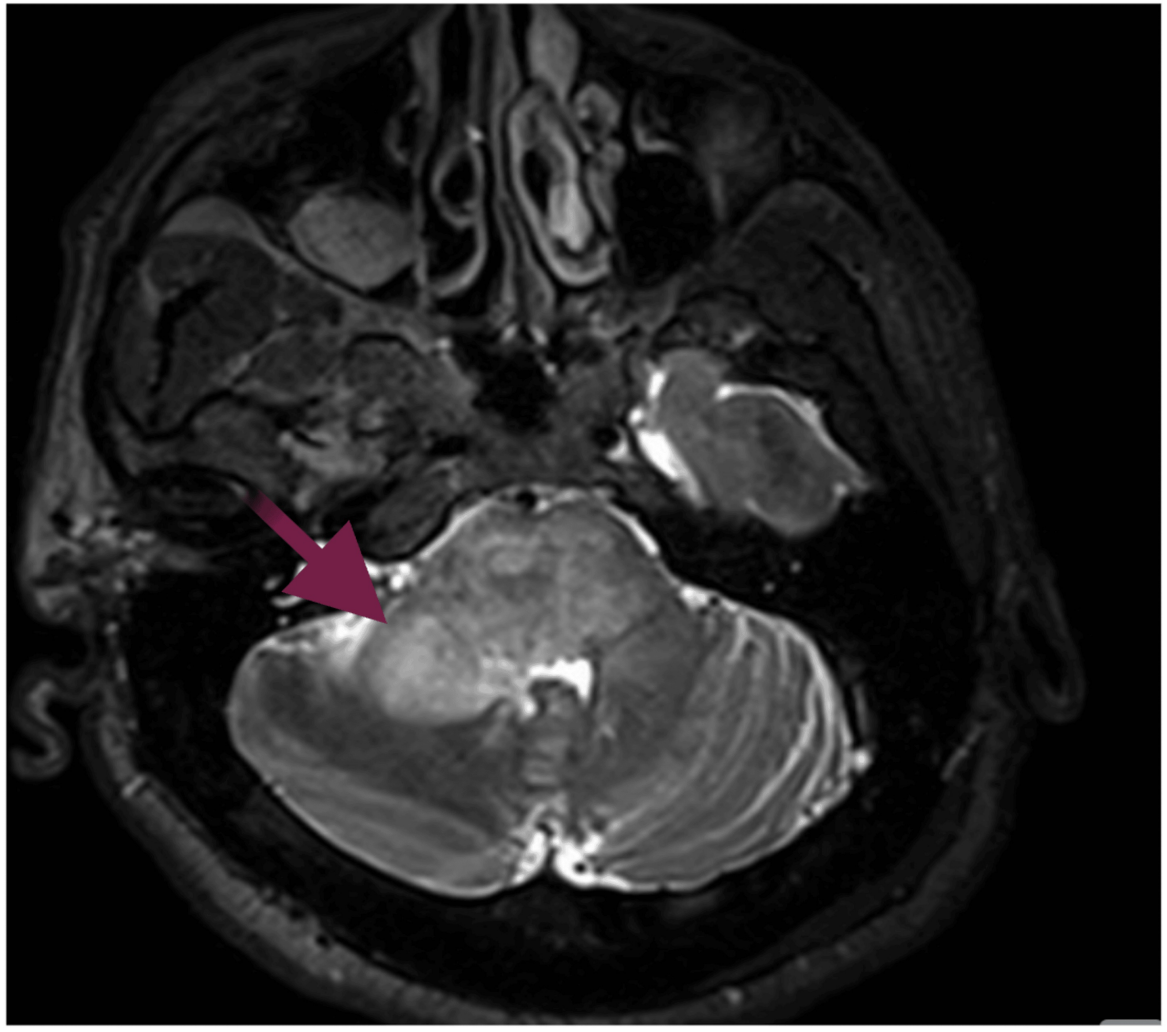
MRI of the brain MRI of the brain demonstrated an enhancing intra-axial mass (red arrow) with central hemorrhagic necrosis in the right pons. This critical finding raised concern for metastasis versus primary high-grade glioma, prompting urgent systemic malignancy workup MRI: magnetic resonance imaging

**Figure 2:**
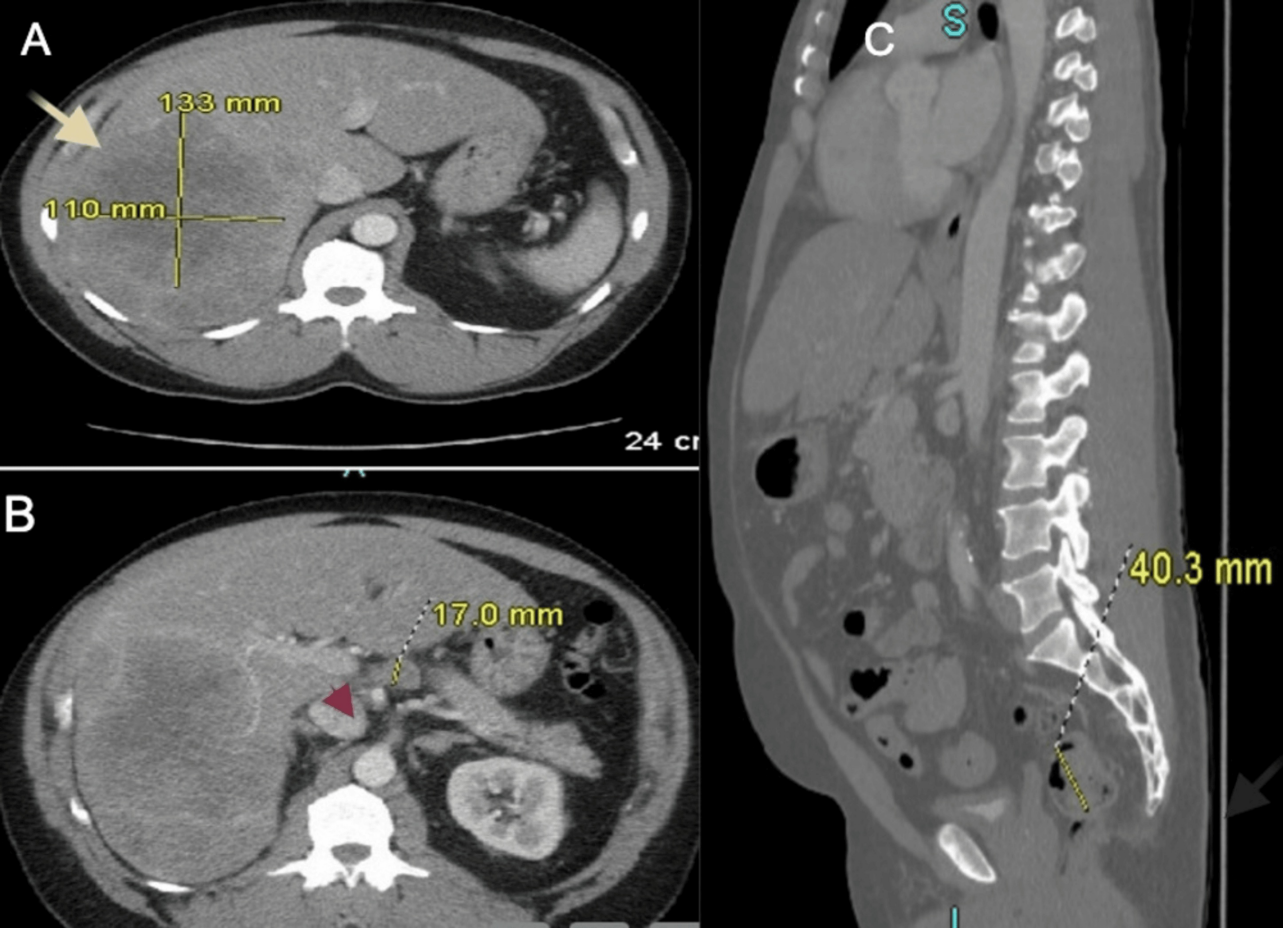
CT scan of the abdomen and pelvis Multiple masses in the right hepatic lobe (A) along with porta hepatis lymphadenopathy (B), both highly suggestive of metastatic disease. Findings in the rectum raised concerns for eccentric mural thickening and nodularity extending to the rectosigmoid junction (C) CT: computed tomography

**Figure 3:**
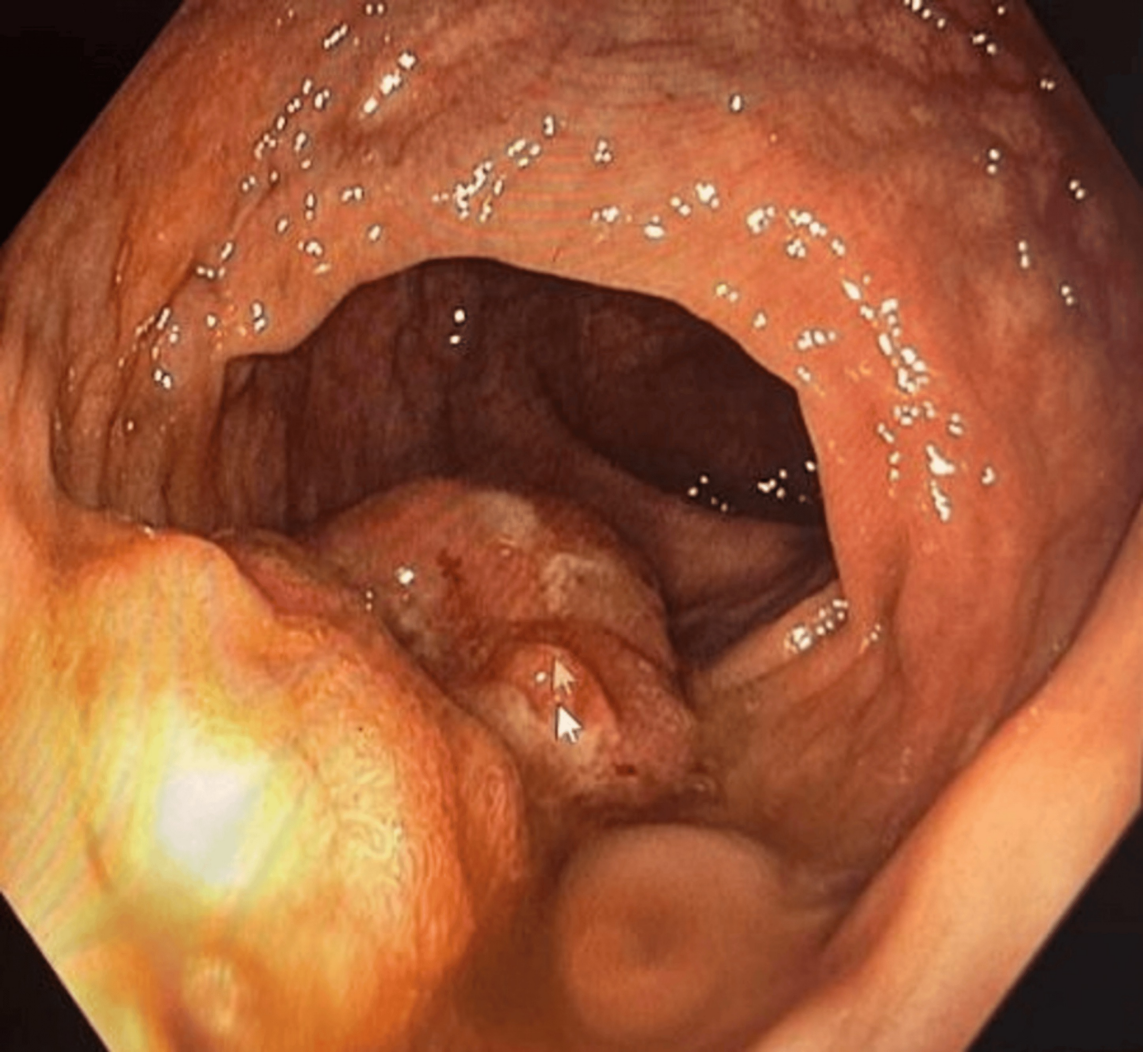
Colonoscopy revealing a rectal mass Visualized rectal mass (arrow) - direct visualization confirming lesion requiring biopsy

A 53-year-old male with a history of asthma presented with progressively worsening headaches over several weeks. The associated symptoms included nausea, dizziness, lightheadedness, and intermittent numbness and tingling on the left side. He denied any weight loss. A CT scan of the head revealed a mass in the right cerebello-pontine angle with surrounding vasogenic edema. MRI of the brain demonstrated an enhancing intra-axial mass with central hemorrhagic necrosis centered in the right pons, concerning for a primary high-grade glioma versus metastasis (Figure 1). A CT scan of the abdomen and pelvis showed multiple masses in the right hepatic lobe (Figure 2A), along with porta hepatis lymphadenopathy (Figure 2B), both highly suggestive of metastatic disease. Findings in the rectum raised concern for eccentric mural thickening and nodularity extending to the rectosigmoid junction (Figure 2C). Colonoscopy confirmed a rectal mass (Figure 3).

A biopsy of the pontine lesion was considered but ultimately not attempted due to the high procedural risks associated with its location. Metastatic disease was favored clinically given the presence of multiple hepatic lesions and extensive lymphadenopathy, findings that strongly suggested systemic metastatic malignancy rather than isolated primary glioblastoma.

**Figure 4:**
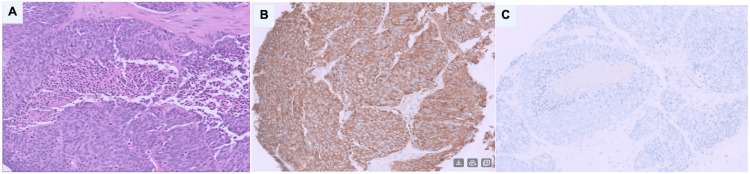
Liver biopsy A: Hematoxylin and eosin stain with intermediate magnification revealed large-cell neuroendocrine carcinoma, with tumor cells that showed large size and eosinophilic cytoplasm B: Immunohistochemistry for synaptophysin with high magnification: immunostains were performed, and the tumor cells were strongly positive for synaptophysin and CD56 (neuroendocrine markers) and negative for the additional neuroendocrine marker chromogranin C: Immunohistochemistry with high magnification: the tumor cells were also negative for CDX2 and CK20 (colonic adenocarcinoma markers), CK7 (lung/pancreaticobiliary marker), and TTF-1 (lung marker)

Liver biopsy revealed large-cell NEC, with tumor cells that showed large size and eosinophilic cytoplasm (Figure 4A). Immunostains were performed, and the tumor cells were strongly positive for synaptophysin and CD56 (neuroendocrine markers) (Figure 4B) and negative for the additional neuroendocrine marker chromogranin. The tumor cells were also negative for CDX2 and CK20 (colonic adenocarcinoma markers), CK7 (lung/pancreaticobiliary marker), and TTF-1 (lung marker) (Figure 4C). The Ki-67 proliferation index, essential for grading NEC and guiding therapy, was unavailable due to limited biopsy material, limiting full characterization of tumor aggressiveness.

**Figure 5:**
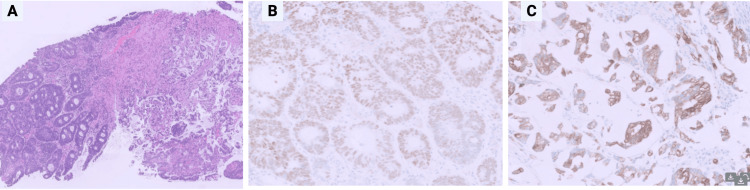
Rectal biopsy A: Hematoxylin and eosin stain with intermediate magnification: moderately differentiated adenocarcinoma with focal mucinous differentiation and glandular architecture B: Immunohistochemistry with high magnification: immunostains revealed that the tumor cells were positive for CDX2 (intestinal marker) and negative for CK20 and CK7 C: Immunohistochemistry for synaptophysin with high magnification: immunostain for synaptophysin was mostly negative, with only focal positive staining in the mucinous adenocarcinoma, which was insufficient to support neuroendocrine differentiation

Rectal biopsy showed invasive, moderately differentiated colorectal adenocarcinoma with focal mucinous differentiation (Figure 5A). Immunostains revealed that the tumor cells were positive for CDX2 (intestinal marker) (Figure 5B) and negative for CK20 and CK7. Immunostain for CD56 was essentially negative. Immunostain for synaptophysin was mostly negative, with only focal positive staining in the mucinous adenocarcinoma (Figure 5C). Morphologically, there was no neuroendocrine differentiation. Immunohistochemistry for mismatch repair (MMR) proteins was performed (block C1), and the tumor cells showed intact nuclear staining for MLH-1, PMS-2, MSH-6, and MSH-2. This pattern indicated an intact mismatch repair function and did not support microsatellite instability (MSS/MSI-L).

The oncology board diagnosed the patient with stage IV NEC of unknown primary origin. Differential considerations included two distinct primary malignancies (rectal adenocarcinoma and hepatic NEC), a mixed neuroendocrine-adenocarcinoma tumor, or adenocarcinoma with neuroendocrine differentiation following metastasis to the liver. The patient received one cycle of chemotherapy with etoposide and carboplatin, along with dexamethasone for brain edema related to pontine metastasis. In the weeks following chemotherapy, the patient developed generalized weakness, which led to admission to a skilled nursing facility. It was anticipated that the patient would undergo a course of radiotherapy for the pontine metastasis. As per the last available follow-up information, the patient remained in the skilled nursing facility with persistent neurologic symptoms, and there was no documentation confirming the initiation of radiotherapy.

## Discussion

Our case was distinctive in both presentation and diagnostic complexity, with pathology identifying hepatic large-cell NEC alongside rectal adenocarcinoma. Differential diagnoses included two separate primary tumors (rectal adenocarcinoma and hepatic NEC), a mixed neuroendocrine-adenocarcinoma tumor, or a rectal adenocarcinoma exhibiting neuroendocrine differentiation after metastasizing to the liver.

Mixed adenoneuroendocrine carcinoma (MANEC) is a rare subtype of colorectal cancer. The WHO defined and reclassified this carcinoma in 2019 as the coexistence of adenocarcinomatous and neuroendocrine components, each comprising at least 30% of the tumor mass. It frequently presents in advanced stages with nodal and distant metastases [3]. While our patient’s hepatic NEC and rectal adenocarcinoma were morphologically and immunohistochemically distinct, the possibility of a MANEC could not be entirely excluded without molecular profiling. A comparable case with diagnostic challenges has been reported by Ito et al., involving a 39-year-old male with hepatic metastases and a transverse colon tumor who was initially thought to have NEC of the colon and multiple hepatic metastases. Subsequent histopathologic evaluation revealed the tumor to be a MANEC, with both components identified in the primary and metastatic sites, a diagnosis only confirmed after exhaustive sampling [4]. The primary treatment for MANEC involves surgical removal of the tumor and any metastases, with adjuvant platinum-based chemotherapy often used due to its aggressive behavior and high risk of recurrence [5].

Multiple primary malignancies occur in approximately 3.7% of cancer patients and entail more than one cancer, either simultaneously (synchronous) or at different times (metachronous), in the same individual. Cancers are considered multiple primaries if they originate in different anatomical sites or have distinct histological or morphological characteristics. This classification helps distinguish true multiple primaries from multifocal/multicentric tumors or metastases. A cancer is termed the index cancer when there is no previous history of invasive malignancy. Recent studies have shown an upward trend in prevalence, with some reporting rates as high as 17% [6]. This rise is likely linked to improved diagnostic methods, extended patient survival, and a larger number of individuals living beyond cancer diagnosis [7]. Importantly, synchronous primary tumors, especially those with histologically discordant features such as NEC and adenocarcinoma, are particularly rare and diagnostically complex. When rectal adenocarcinoma and hepatic NEC represent two independent primaries, each should be staged and managed per NCCN guidelines [8].

Colorectal adenocarcinoma with neuroendocrine differentiation might suggest that the primary colorectal tumor spread to the liver and later exhibited neuroendocrine characteristics. Although this occurrence is rare and the literature on it is limited, some studies have noted a high rate of differentiation in metastatic colorectal carcinoma cases [9]. However, it remains unclear whether this differentiation originated in the colorectal region or occurred after metastasis to the liver.

Several diagnostic limitations were evident in this case. Firstly, molecular profiling or genomic analysis, including next-generation sequencing, was not performed, preventing definitive clarification of the clonality and primary tumor relationships. Second, the Ki-67 proliferation index, a critical marker for grading neuroendocrine carcinomas and guiding therapy, was not available due to specimen constraints. Third, somatostatin receptor imaging (e.g., DOTATE PET/CT) was not performed, which could have helped further characterize the neuroendocrine component and guided potential peptide receptor radionuclide therapy. Recognizing these limitations explicitly is important, as they directly impact the diagnostic precision and subsequent therapeutic strategies.

## Conclusions

This report adds to the limited literature on synchronous NEC and adenocarcinoma, demonstrating how metastatic patterns can obscure tumor origins. The rising incidence of multiple primary malignancies, attributed to improved diagnostics and survivorship, necessitates updated guidelines for staging and therapy. Critically, molecular profiling (e.g., next-generation sequencing) must be pursued in similar cases to definitively distinguish dual primaries from clonal evolution, enabling biologically guided personalized therapy.
